# Assessment of teaching and learning environment in Southwest China: A cross-sectional study on medical intern satisfaction

**DOI:** 10.1097/MD.0000000000050416

**Published:** 2026-08-28

**Authors:** Xingning Mao, Li Shao, Haiyan Yao, Hua Liang, Yuning Wei, Jie Yu, Liujuan Qin, Yanting Zhou

**Affiliations:** a Academic Affairs Office of Guangxi Medical University, Nanning, People’s Republic of China; b Undergraduate Student in Clinical Medicine, Guangxi Medical University, Nanning, People’s Republic of China.

**Keywords:** cross-sectional study, educational experience enhancement, intern satisfaction, medical education, teaching and learning environment

## Abstract

The teaching and learning environment (TLE) plays a pivotal role in medical education by influencing students’ professional development and quality of patient care. However, satisfaction levels among students varied within the TLE. This study aimed to assess TLE in medical education in southwest China. A cross-sectional study was conducted from April to May 2020 in southwest China to assess TLE among clinical medical interns across 17 hospitals, with data collected via an online questionnaire focusing on demographics, TLE perception, educational guidance, and overall satisfaction. Ordinal logistic regression was performed to identify factors that correlated with overall satisfaction. Among the 834 medical interns, 74.10% reported satisfaction with their teaching hospital, with the highest satisfaction observed in medical record writing and the lowest in living conditions. Ordinal logistic regression identified several predictors of higher satisfaction, including satisfaction with teaching rooms, teaching management staff, and medical procedures, as well as a sense of responsibility instilled by teaching preceptors and managing an average of 1 to 2 or 3 to 4 beds. Notably, attending mini-lectures at a bimonthly frequency was significantly correlated with increased satisfaction. This study highlights the importance of teaching room quality, the responsibility of teaching preceptors, medical procedures, and patient bed management, as well as the frequency of mini-lectures, as key factors influencing interns’ satisfaction. These findings provide valuable insights for medical education institutions seeking to enhance their clinical training experience and the overall satisfaction of medical interns.

## 1. Introduction

The teaching and learning environment (TLE) constitutes a fundamental pillar of medical education and plays a pivotal role in shaping educational experiences and outcomes for future healthcare professionals.^[1]^ A well-crafted TLE is essential for fostering a culture of inquiry, reflection, and clinical excellence.^[2]^ It encompasses not only the physical infrastructure and available resources but also the educational climate, which includes attitudes, values, and behaviors exemplified by faculty and staff.^[3]^ TLE significantly impacts students’ professional development and clinical competencies, ultimately influencing the quality of patient care they deliver after graduation.^[4]^

Despite the well-established importance of a conducive TLE, satisfaction levels among medical students vary considerably.^[5,6]^ While some students flourish in supportive and intellectually stimulating environments, others may encounter challenges, such as overcrowded classrooms, insufficient resources, or limited faculty support. Studies have revealed a range of satisfaction levels with implications for student engagement, academic performance, and mental well-being.^[7]^ This variability highlights the need for a nuanced understanding of the factors that foster positive TLE as well as the identification of areas requiring targeted attention and improvement.

The existing literature highlights several prevalent issues within TLE, including insufficient integration of technology, reliance on outdated pedagogical approaches, and a disconnect between educational theory and practice.^[8,9]^ These issues can impede the development of competent healthcare providers. Therefore, this study aims to investigate the current state of TLE in medical education in southwest China and to identify key areas of concern that could be addressed to enhance the educational experience of medical students.

## 2. Materials and methods

### 2.1. Study design

This cross-sectional study was performed from April 2020 to May 2020 in Southwest China. Participants included clinical medical interns who had been practicing in 17 teaching hospitals in 7 cities for approximately 6 months. Among these hospitals, 7 were provincial-level and 10 were municipal-level institutions. Convenience sampling was also conducted. Informed consent forms and questionnaires were distributed to the directors of the teaching management departments at the aforementioned hospitals, who were instructed to disseminate the survey to their medical interns. Both the informed consent form and questionnaire were completed online. After reviewing the informed consent form, which outlines the purpose, methodology, and content of the study, as well as the rights and benefits of participation, medical interns would indicate their willingness to participate in the research by clicking “Agree” to proceed with the survey. The questionnaire was self-administered and designed to be completed in approximately 5 minutes. The survey was conducted in accordance with the Declaration of Helsinki and was approved by the Institutional Ethics Committee of Guangxi Medical University.

### 2.2. Questionnaire

The questionnaire was designed by our teaching management team according to a document released by the Ministry of Education of the People’s Republic of China,^[10]^ which aimed at evaluating the conditions and implementation of teaching in Clinical Teaching Bases from the perspective of students, and analyzing the satisfaction of interns with the hospital’s teaching environment. The domains – including teaching conditions (e.g., classrooms, clinical skills rooms), faculty resources (e.g., teaching supervisors’ sense of responsibility, professional title composition), and teaching implementation (e.g., frequency of mini-lectures, case discussions) – underwent rigorous review and validation by medical education experts. The questionnaire consists of 4 parts: demographic information (gender, age, residence, ethnicity, only children, grade, class, teaching hospital), perception of TLE, teaching and learning guidance, and overall satisfaction. Perception of TLE consisted of 9 questions to evaluate medical interns’ perceptions of the basic conditions, teaching and learning management, and clinical practice of the teaching hospital. The questions can be answered with “satisfied” or “dissatisfied.” Teaching and learning guidance consisted of 8 questions to evaluate the teaching preceptor, interns’ average beds under management, and frequency of teaching activities. Finally, they were asked, ‘How satisfied are you with this hospital overall?’ to assess interns’ overall satisfaction with the hospital. The interns can respond with “satisfied,” “neutral, or “dissatisfied.” The quesitonnaire demonstrated acceptable reliability (Cronbach’s α = 0.780) and validity (Kaiser–Meyer–Olkin = 0.805, Bartlett’s test *P* = .000).

### 2.3. Statistical analyses

SPSS 25.0 (IBM, Chicago) was used to analyze the collected data. Categorical variables are described using frequencies and percentages. Ordinal Logistic regression analysis was conducted to investigate the factors associated with interns’ overall satisfaction with the teaching hospitals. The parallel-lines test was conducted to assess whether the proportional odds assumption held, and Pearson’s chi-square and Deviance chi-square statistics were used to evaluate the model fit. Statistical *P* was set at less than .05.

## 3. Results

A total of 1449 clinical interns were invited to participate in the survey, and 858 completed the questionnaire, yielding a response rate of 59.21% (858/1449). During the process of data organization and analysis, the data from 24 interns were deleted by listwise deletion due to incomplete data. Therefore, we ultimately analyzed the data of 834 interns. As shown in Table 1, 834 medical interns were included in the study; 312 (37.41%) were males and 522 (62.59%) were females. The age of the interns ranged from 21 to 23 years, with a mean of 22.42 ± 0.61 years. A total of 331 interns lived in cities (39.69%), and 503 interns lived in rural areas (60.31%). All interns came from Han (72.66%), Zhuang (22.3%), and other ethnic minorities (5.04%). The data also showed that 199 (23.86%) interns came from single-child families, while 635 (76.14%) came from families with multiple children. Regarding the overall satisfaction of all interns, 618 (74.10%) were satisfied with the teaching hospital. There were no significant differences in sex, age, residence, ethnicity, only children, and overall satisfaction (all *P* > .05).

**Table 1:** Correlation between the characteristics of students and overall satisfaction.

Items	n = 834	Satisfied	Neutral	Dissatisfied	χ^2^	*P*
		(n = 618, 74.10)	(n = 201, 24.10)	(n = 15, 1.80)		
Gender					0.517	.772
Male	312 (37.41)	235 (75.32)	71 (22.76)	6 (1.92)		
Female	522 (62.59)	383 (73.37)	130 (24.9)	9 (1.72)		
Age	22.42 ± 0.61	22.42 ± 0.61	22.4 ± 0.59	22.20 ± 0.56	1.261	.284
Residence					1.393	.498
Urban	331 (39.69)	241 (72.81)	82 (24.77)	8 (2.42)		
Rural	503 (60.31)	377 (74.95)	119 (23.66)	7 (1.39)		
Ethnicity					5.052	.282
The Han	606 (72.66)	459 (75.74)	139 (22.94)	8 (1.32)		
The Zhuang	186 (22.3)	130 (69.89)	50 (26.88)	6 (3.23)		
The Others	42 (5.04)	29 (69.05)	12 (28.57)	1 (2.38)		
Only children					0.768	.681
Yes	199 (23.86)	147 (73.87)	47 (23.62)	5 (2.51)		
No	635 (76.14)	471 (74.17)	154 (24.25)	10 (1.57)		

**Figure 1.**
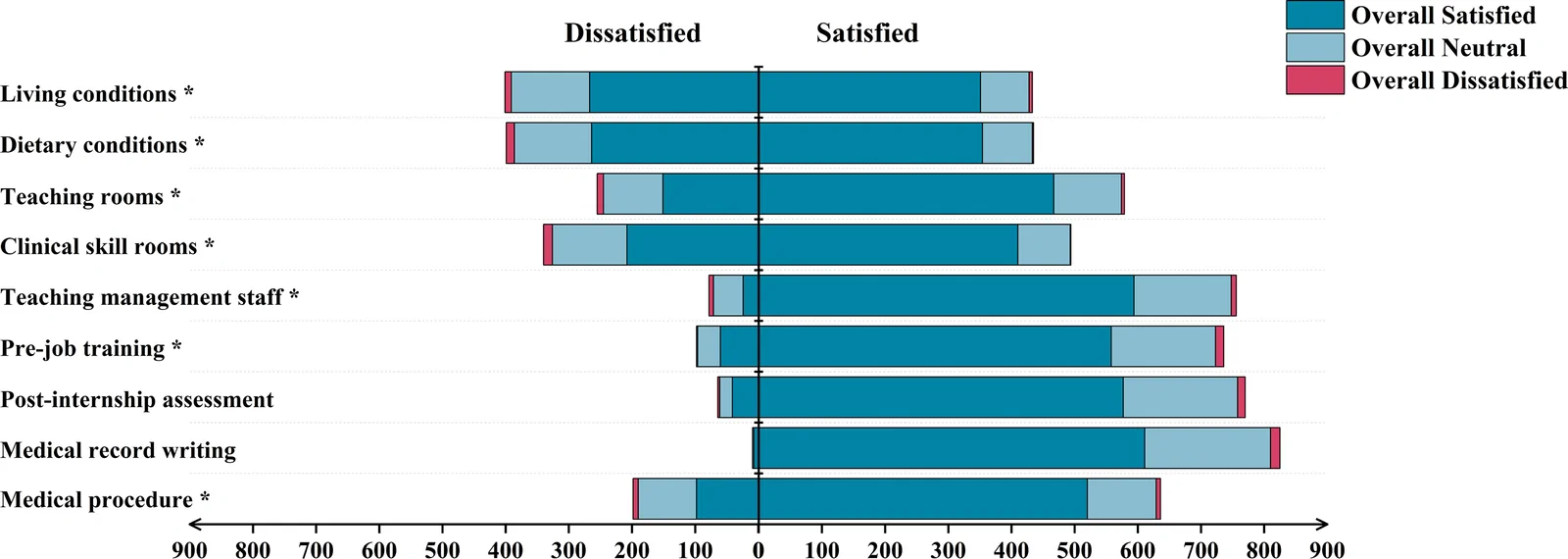
Correlation between interns’ perception of TLE and overall satisfaction. The horizontal axis displays dissatisfaction (left) and satisfaction (right) frequencies across 9 factors of TLE, with color-coding indicating overall satisfaction categories. Color segments within bars correspond to: Blue indicates “Overall Satisfied,” Light blue indicates “Overall Neutral,” Red indicates “Overall Dissatisfied.” *P < .05. TLE = teaching and learning environment.

Figure 1 shows that the medical interns’ perceptions of living conditions, dietary conditions, teaching rooms, clinical skill rooms, teaching management staff, prejob training, and medical procedures were positively associated with overall satisfaction (all *P* < .01), and those who were satisfied with the above conditions were more satisfied with the teaching hospital. Medical interns had the highest satisfaction with medical record writing (825, 98.92%) and lowest satisfaction with living conditions (433, 51.92%). More detailed information on the correlation between interns’ perception of TLE and overall satisfaction in the Table S1, Supplemental Digital Content 1.

**Figure 2.**
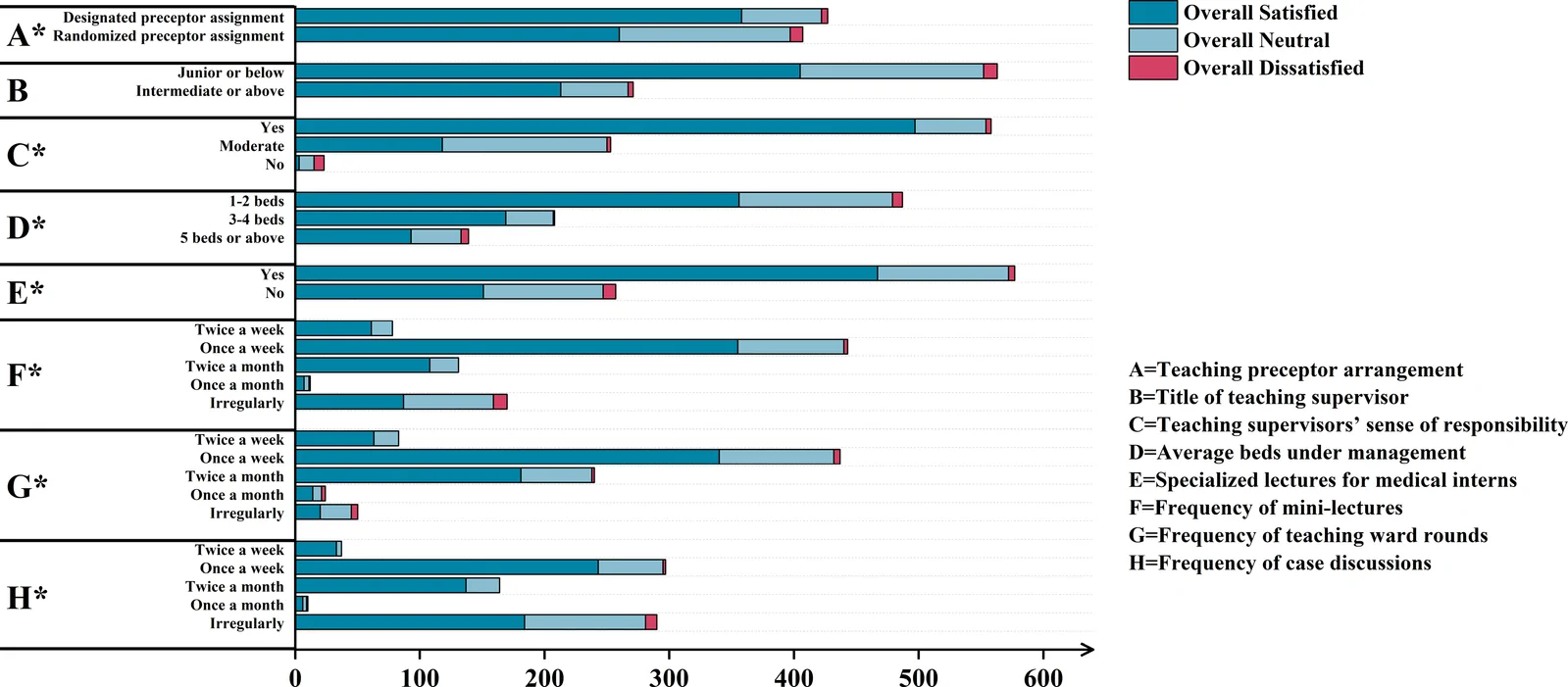
Correlation between teaching and learning guidance and overall satisfaction. The figure displays the correlation between 8 factors of teaching and learning guidance and overall satisfaction in detail, with color-coding indicating overall satisfaction categories. Color segments within bars correspond to: Blue indicates “Overall Satisfied,” Light blue indicates “Overall Neutral,” Red indicates “Overall Dissatisfied.” *P < .05.

Figure 2 shows the correlation between teaching and learning guidance and overall satisfaction. Specialized lectures for medical interns, teaching preceptor arrangement, teaching preceptors’ sense of responsibility, average beds under management, frequency of mini-lectures, frequency of teaching ward rounds, and frequency of case discussions were associated with the overall satisfaction (all *P* < .001). 407 (48.80%) medical interns reported that their preceptors were assigned randomly. 558 (66.91%) reported that their teaching preceptors had a sense of responsibility. 487 (58.39%) and 208 (24.94%) reported that their average beds under management were 1 to 2 beds and 3 to 4 beds, respectively. 577 (69.18%) reported that there were specialized lectures for them. Most interns reported that the frequencies of mini-lectures, teaching ward rounds, and case discussions were once a week or twice a month. The aforementioned variables were related to a higher overall satisfaction level. More information can be found in the Table S2, Supplemental Digital Content 2.

We included variables with statistically significant differences in the ordinal logistic regression to identify predictors of interns’ overall satisfaction with teaching hospitals (Table 2). The parallel-lines test indicated that the proportional odds assumption held for the model (*P* = .789), supporting the use of ordinal logistic regression for this study. The Model Fitting Information indicated that the regression model was statistically significant (*P* < .001). Model fit was assessed using Pearson’s chi-square and Deviance chi-square statistics; both were nonsignificant (*P* > .05), indicating adequate model specification and fit. The final model retained 8 predictors. Those who were satisfied with the teaching rooms (OR = 1.554, *P* = .048), teaching management staff (OR = 2.067, *P* = .027), and medical procedures (OR = 1.863, *P* = .006) were more likely to report higher overall satisfaction. Teaching preceptors a sense of responsibility was significantly associated with a higher satisfaction level (Moderate: OR 5.362, *P* = .003; Yes: OR = 33.127, *P* < .001). Medical interns who managed an average of 1 to 2 (OR = 1.921, *P *= .013) or 3 to 4 beds (OR = 2.456, *P* = .005) tended to have a higher satisfaction level. The frequency of twice-monthly mini-lectures was associated with a higher level of satisfaction (OR = 2.604, *P* = .013).

**Table 2:** Positive results of ordinal regression analysis with overall satisfaction as the dependent variable.

Items	β	*P* value	Odds ratio	95% CI
Teaching rooms				
Satisfied	0.441	**.048**	1.554	1.004–2.403
Dissatisfied			Reference	
Teaching management staff				
Satisfied	0.726	**.027**	2.067	1.086–3.936
Dissatisfied			Reference	
Teaching supervisors’ sense of responsibility				
Yes	3.500	**<.001**	33.127	10.676–102.793
Moderate	1.679	**.003**	5.362	1.790–16.060
No			Reference	
Medical procedure				
Satisfied	0.622	**.006**	1.863	1.195–2.904
Dissatisfied			Reference	
Average beds under management				
1–2 beds	0.653	**.013**	1.921	1.146–3.219
3–4 beds	0.899	**.005**	2.456	1.313–4.593
5 beds or above			Reference	
Frequency of mini-lectures				
Twice a week	-0.396	.359	0.673	0.289–1.569
Once a week	0.166	.566	1.181	0.669–2.086
Twice a month	0.957	**.013**	2.604	1.228–5.524
Once a month	0.315	.724	1.370	0.238–7.894
Irregularly			Reference	

Values in bold indicate statistically significant differences (*P* < .05).

## 4. Discussion

Our study indicated that 74.10% of medical interns were satisfied with their teaching hospital, reflecting the quality of the education and training they received. Intern satisfaction was strongly associated with several factors, including teaching rooms, teaching management staff, sense of responsibility of teaching preceptors, medical procedures, average number of beds under management, and frequency of mini-lectures. Notably, medical interns expressed the highest satisfaction with medical record writing, which may stem from the high quality of medical record writing. The instructors’ feedback of medical records correcting allows students to directly perceive their learning progress. Moreover, the standardized templates and clear steps can enable students to easily master and complete medical record writing, resulting in less frustration for learners. Medical record writing has great educational value for clinical interns. The experience of medical record writing enables medical students to learn about patient encounters in both inpatient and outpatient care venues, reflect on patient encounters, learn proper documentation skills, and attain a sense of being actively involved in and responsible for the care of patients through medical record writing.^[11]^

Nearly half of the interns reported that their preceptors were randomly assigned and were of junior rank or below. Previous studies have demonstrated that effective mentorship enhances interns’ self-efficacy, professional identity, and well-being, which in turn increases their satisfaction with their training programs.^[12,13]^ A systematic scoping review by Teo et al conceptualizes mentorship as a complex adaptive system (CAS), highlighting the dynamic, evolving, and reciprocal nature of these relationships.^[14]^ Mentorship is not a linear process, but rather 1 that involves multiple agents, such as mentors, mentees, and institutional leaders, interacting within a collaborative network and influencing each other’s behaviors and perspectives. The availability of supportive mentors and preceptors plays a significant role in medical interns’ satisfaction. Specifically, the quality of supervision, accessibility, and level of guidance significantly impact interns’ learning outcomes and overall satisfaction.^[14]^ Given the relatively high proportion of interns reporting junior or randomly assigned preceptors, there remains significant scope to enhance the internship program by strengthening supervision quality and mentorship structures.

Our findings further highlight a robust correlation between medical interns’ satisfaction with medical procedures and their overall internship experience, with notably higher satisfaction among those who found procedural involvement fulfilling. Notably, interns managing a moderate patient load of 1 to 2 or 3 to 4 beds reported elevated satisfaction levels, suggesting that balanced responsibility in patient care positively contributes to their professional development and confidence. Excessive patient assignments can lead to an overworkload and significantly reduce the job satisfaction of physicians,^[15]^ which can also cause burnout and lower interns’ enthusiasm for learning. These insights highlight the imperative for medical education programs to integrate comprehensive procedural learning within a supportive framework and to thoughtfully delegate patient care duties. Moreover, they emphasize the necessity for institutions to actively solicit and incorporate internal feedback to enhance the educational environment and curriculum design.^[16]^ By doing so, teaching hospitals can cultivate a more contented and competent future healthcare workforce committed to delivering high-quality patient care.^[17]^

The positive correlation between satisfaction with teaching rooms and overall satisfaction among medical interns further underscores the critical role of the learning environment in medical education. A well-equipped and comfortable physical setting not only enhances the learning experience but also contributes to psychological comfort, academic performance, and a sense of belonging.^[18]^ This association emphasizes the need for institutions to prioritize infrastructure development, actively engage in continuous improvement based on internal feedback, and foster supportive and inclusive teaching spaces. Furthermore, it suggests that both physical and social dynamics within teaching environments significantly impact the educational outcomes and well-being of medical interns, emphasizing their critical role in achieving excellence in medical education.^[2,19]^

The observed correlation between the bimonthly frequency of mini-lectures and elevated satisfaction levels provides valuable insights into educational preferences that enhance the learning experience. It suggests the necessity of optimizing the frequency of mini-lectures. An insufficient frequency may compromise the systematic integration and timely reinforcement of clinical knowledge for interns, while excessive frequency could encroach upon hands-on learning opportunities and even cause cognitive fatigue among interns. Such scheduling likely optimizes cognitive load management by presenting information in digestible increments, thereby facilitating improved knowledge assimilation and retention.^[20]^ This pacing allows for reinforcement and reflection, potentially fostering greater learner engagement with the material.^[21]^ Interactive and discussion-oriented mini-lectures, when appropriately spaced, offer an effective platform for learning that aligns with students’ cognitive capabilities and preferences.^[22]^ Institutions are encouraged to leverage this feedback to refine pedagogical strategies, ensuring that lecture frequency and format promote a positive and effective learning environment. Further research is warranted to explore how these preferences affect learning outcomes beyond satisfaction, thereby providing a more comprehensive understanding of educational effectiveness.

The relatively low satisfaction rate of medical interns regarding their accommodation and dining conditions (approximately 50%) reveals a critical area for enhancement and underscores the need for a more holistic approach to interns’ well-being. Institutions must ensure that the physical comfort, nutritional requirements, and cultural considerations of interns are adequately addressed to support their health and performance.^[23]^ Recognizing that a supportive environment can enhance academic outcomes, it is imperative for institutions to seek active feedback and implement continuous improvements in living and dining conditions.^[18]^ Moreover, acknowledging the institutional responsibility to provide a conducive living and learning environment, future research and policy development should focus on identifying and addressing specific factors contributing to dissatisfaction. By fostering an inclusive space that caters to the diverse needs of interns, teaching hospitals can significantly enhance the overall success and well-being of medical interns, thereby promoting positive and enriching educational experiences.^[24]^

Although this study provides valuable insights into the factors contributing to medical interns’ satisfaction with their teaching hospital, several limitations must be acknowledged. First, the cross-sectional design restricts the ability to infer the causality or directionality of observed relationships. Second, while the moderate response rate (59.21%) was within acceptable limits for survey research, it remained a primary study limitation due to potential nonresponse bias, particularly as systematic differences between respondents and nonrespondents could introduce selection bias. Although the samples included in the research cover 17 teaching hospitals across 7 cities in Southwest China and share homogeneous characteristics with the broader population of medical interns in Southwest China, the representativeness of the samples should be taken into consideration as a limitation for this study. In addition, convenience sampling was conducted due to the unique challenges of recruiting medical interns (e.g., fixed clinical rotation schedules, concentrated in teaching hospitals) in this research. It lacked randomness and may potentially compromise representativeness, while being convenient and cost-effective. Finally, although several factors positively associated with overall satisfaction were identified, reliance on self-reported data might be vulnerable to social desirability and recall bias, potentially compromising response accuracy.

## 5. Conclusions

With 74.10% of medical interns expressing satisfaction, several key predictors of overall satisfaction were identified, including the quality of teaching rooms, teaching preceptors’ sense of responsibility, medical procedures, average beds under management, and frequency of mini-lectures. These findings provide valuable guidance for institutions seeking to enhance both the educational experience and the overall satisfaction of medical interns.

## Acknowledgments

We offer special gratitude to the participants of this study for their support.

## Author contributions

**Data curation:** Li Shao, Haiyan Yao, Hua Liang, Yuning Wei.

**Investigation:** Li Shao, Haiyan Yao, Hua Liang, Yuning Wei, Jie Yu, Liujuan Qin.

**Funding acquisition:** Yanting Zhou.

**Project administration:** Yanting Zhou.

**Writing – original draft:** Xingning Mao.

**Writing – review & editing:** Xingning Mao.




