# Supplementary material for: Assessment of teaching and learning environment in Southwest China: A cross-sectional study on medical intern satisfaction
Source: Medicine (Baltimore). 2026 Aug 28;105(35):e50416. doi: 10.1097/MD.0000000000050416 (PMC13529160; doi:10.1097/MD.0000000000050416)
Supplement: Supplementary file 1 [file medi-105-e50416-s001.doc]

| Table S1 Correlation between interns’ perception of TLE and overall satisfaction | | | | | | |
| --- | --- | --- | --- | --- | --- | --- |
| Items | n=834 | Satisfied (n=618, 74.10) | Neutral (n=201, 24.10) | Dissatisfied (n=15, 1.80) | χ^2^ | *p* |
| Living conditions |  |  |  |  | 22.880 | <0.001 |
| Satisfied | 433(51.92) | 351(81.06) | 77(17.78) | 5(1.15) |  |  |
| Dissatisfied | 401(48.08) | 267(66.58) | 124(30.92) | 10(2.49) |  |  |
| Dietary conditions |  |  |  |  | 28.872 | <0.001 |
| Satisfied | 435(52.16) | 354(81.38) | 79(18.16) | 2(0.46) |  |  |
| Dissatisfied | 399(47.84) | 264(66.17) | 122(30.58) | 13(3.26) |  |  |
| Teaching rooms |  |  |  |  | 45.009 | <0.001 |
| Satisfied | 579(69.42) | 467(80.66) | 107(18.48) | 5(0.86) |  |  |
| Dissatisfied | 255(30.58) | 151(59.22) | 94(36.86) | 10(3.92) |  |  |
| Clinical skill rooms |  |  |  |  | 56.890 | <0.001 |
| Satisfied | 494(59.23) | 410(83.00) | 83(16.80) | 1(0.20) |  |  |
| Dissatisfied | 340(40.77) | 208(61.18) | 118(34.71) | 14(4.12) |  |  |
| Teaching management staff |  |  |  |  | 93.111 | <0.001 |
| Satisfied | 756(90.65) | 594(78.57) | 154(20.37) | 8(1.06) |  |  |
| Dissatisfied | 78(9.35) | 24(30.77) | 47(60.26) | 7(8.97) |  |  |
| Pre-job training |  |  |  |  | 9.876 | 0.007 |
| Satisfied | 736(88.25) | 558(75.82) | 165(22.42) | 13(1.77) |  |  |
| Dissatisfied | 98(11.75) | 60(61.22) | 36(36.73) | 2(2.04) |  |  |
| Post-internship assessment |  |  |  |  | 5.629 | 0.060 |
| Satisfied | 770(92.33) | 577(74.94) | 181(23.51) | 12(1.56) |  |  |
| Dissatisfied | 64(7.67) | 41(64.06) | 20(31.25) | 3(4.69) |  |  |
| Medical record writing |  |  |  |  | 0.194 | 0.908 |
| Satisfied | 825(98.92) | 611(74.06) | 199(24.12) | 15(1.82) |  |  |
| Dissatisfied | 9(1.08) | 7(77.78) | 2(22.22) | 0(0.00) |  |  |
| Medical procedure |  |  |  |  | 82.351 | <0.001 |
| Satisfied | 636(76.26) | 520(81.76) | 109(17.14) | 7(1.10) |  |  |
| Dissatisfied | 198(23.74) | 98(49.49) | 92(46.46) | 8(4.04) |  |  |
