# Supplementary material for: Assessment of teaching and learning environment in Southwest China: A cross-sectional study on medical intern satisfaction
Source: Medicine (Baltimore). 2026 Aug 28;105(35):e50416. doi: 10.1097/MD.0000000000050416 (PMC13529160; doi:10.1097/MD.0000000000050416)
Supplement: Supplementary file 2 [file medi-105-e50416-s002.doc]

| Table S2 Correlation between teaching and learning guidance and overall satisfaction | | | | | | |
| --- | --- | --- | --- | --- | --- | --- |
| Items | n=834 | Satisfied (n=618, 74.10) | Neutral (n=201, 24.10) | Dissatisfied (n=15, 1.80) | χ^2^ | *p* |
| Teaching supervisor arrangement |  |  |  |  | 43.265 | <0.001 |
| Designated preceptor assignment | 427(51.20) | 358(83.84) | 64(14.99) | 5(1.17) |  |  |
| Randomized preceptor assignment | 407(48.80) | 260(63.88) | 137(33.66) | 10(2.46) |  |  |
| Title of teaching supervisor |  |  |  |  | 4.231 | 0.121 |
| Junior or below | 563(67.51) | 405(71.94) | 147(26.11) | 11(1.95) |  |  |
| Intermediate or above | 271(32.49) | 213(78.60) | 54(19.93) | 4(1.48) |  |  |
| Teaching supervisors’ sense of responsibility |  |  |  |  | 332.367 | <0.001 |
| Yes | 558(66.91) | 497(89.07) | 57(10.22) | 4(0.72) |  |  |
| Moderate | 253(30.34) | 118(46.64) | 132(52.17) | 3(1.19) |  |  |
| No | 23(2.76) | 3(13.04) | 12(52.17) | 8(34.78) |  |  |
| Average beds under management |  |  |  |  | 13.912 | 0.008 |
| 1-2 beds | 487(58.39) | 356(73.10) | 123(25.26) | 8(1.64) |  |  |
| 3-4 beds | 208(24.94) | 169(81.25) | 38(18.27) | 1(0.48) |  |  |
| 5 beds or above | 139(16.67) | 93(66.91) | 40(28.78) | 6(4.32) |  |  |
| Specialized lectures for medical interns |  |  |  |  | 47.922 | <0.001 |
| Yes | 577(69.18) | 467(80.94) | 105(18.20) | 5(0.87) |  |  |
| No | 257(30.82) | 151(58.75) | 96(37.35) | 10(3.89) |  |  |
| Frequency of mini-lectures |  |  |  |  | 77.241 | <0.001 |
| Twice a week | 78(9.35) | 61(78.21) | 17(21.79) | 0(0.00) |  |  |
| Once a week | 443(53.12) | 355(80.14) | 85(19.19) | 3(0.68) |  |  |
| Twice a month | 131(15.71) | 108(82.44) | 23(17.56) | 0(0.00) |  |  |
| Once a month | 12(1.44) | 7(58.33) | 4(33.33) | 1(8.33) |  |  |
| Irregularly | 170(20.38) | 87(51.18) | 72(42.35) | 11(6.47) |  |  |
| Frequency of teaching ward rounds |  |  |  |  | 63.179 | <0.001 |
| Twice a week | 83(9.95) | 63(75.90) | 20(24.10) | 0(0.00) |  |  |
| Once a week | 437(52.40) | 340(77.80) | 92(21.05) | 5(1.14) |  |  |
| Twice a month | 240(28.78) | 181(75.42) | 57(23.75) | 2(0.83) |  |  |
| Once a month | 24(2.88) | 14(58.33) | 7(29.17) | 3(12.50) |  |  |
| Irregularly | 50(6.00) | 20(40.00) | 25(50.00) | 5(10.00) |  |  |
| Frequency of case discussions |  |  |  |  |  |  |
| Twice a week | 37(4.64) | 33(89.19) | 4(10.81) | 0(0.00) | 47.276 | <0.001 |
| Once a week | 297(37.22) | 243(81.82) | 52(17.51) | 2(0.67) |  |  |
| Twice a month | 164(20.55) | 137(83.54) | 27(16.46) | 0(0.00) |  |  |
| Once a month | 10(1.25) | 6(60.00) | 3(30.00) | 1(10.00) |  |  |
| Irregularly | 290(36.34) | 184(63.45) | 97(33.45) | 9(3.10) |  |  |
